# Building oral health research infrastructure: the first national oral health survey of Rwanda

**DOI:** 10.1080/16549716.2018.1477249

**Published:** 2018-06-04

**Authors:** John P. Morgan, Moses Isyagi, Joseph Ntaganira, Agnes Gatarayiha, Sarah E. Pagni, Tamar C. Roomian, Matthew Finkelman, Jane E. M. Steffensen, Jane R. Barrow, Chrispinus H. Mumena, Donna M. Hackley

**Affiliations:** a Department of Public Health and Community Service, Tufts University School of Dental Medicine, Boston, USA; b Department of Oral Medicine and Oral Surgery, School of Dentistry, University of Rwanda, Kigali, Rwanda; c School of Public Health, University of Rwanda, Kigali, Rwanda; d Department of Community and Preventive Dentistry, School of Dentistry, University of Rwanda, Kigali, Rwanda; e Division of Biostatistics and Experimental Design, Tufts University School of Dental Medicine, Boston, USA; f Department of Oral Health Policy and Epidemiology, Harvard School of Dental Medicine; g School of Dentistry, University of Rwanda, Kigali, Rwanda; h Department of Preventive and Community Dentistry, University of Rwanda School of Dentistry, Kigali, Rwanda

**Keywords:** Global health, oral health, oral health research infrastructure, electronic survey design, capacity building

## Abstract

**Background**: Oral health affects quality of life and is linked to overall health. Enhanced oral health research is needed in low- and middle-income countries to develop strategies that reduce the burden of oral disease, improve oral health and inform oral health workforce and infrastructure development decisions.

**Objective**: To implement the first National Oral Health Survey of Rwanda to assess the oral disease burden and inform oral health promotion strategies.

**Methods**: In this cross-sectional study, sample size and site selection were based on the World Health Organization (WHO) Oral Health Surveys Pathfinder stratified cluster methodologies. Randomly selected 15 sites included 2 in the capital city, 2 other urban centers and 11 rural locations representing all provinces and rural/urban population distribution. A minimum of 125 individuals from each of 5 age groups were included at each site. A Computer Assisted Personal Instrument (CAPI) was developed to administer the study instrument.

**Results**: Nearly two-thirds (64.9%) of the 2097 participants had caries experience and 54.3% had untreated caries. Among adults 20 years of age and older, 32.4% had substantial oral debris and 60.0% had calculus. A majority (70.6%) had never visited an oral health provider. Quality-of-life challenges due to oral diseases/conditions including pain, difficulty chewing, self-consciousness, and difficulty participating in usual activities was reported at 63.9%, 42.2% 36.2%, 35.4% respectively.

**Conclusion**: The first National Oral Health Survey of Rwanda was a collaboration of the Ministry of Health of Rwanda, the University of Rwanda Schools of Dentistry and Public Health, the Rwanda Dental Surgeons and Dental (Therapists) Associations, and Tufts University and Harvard University Schools of Dental Medicine. The international effort contributed to building oral health research capacity and resulted in a national oral health database of oral disease burden. This information is essential for developing oral disease prevention and management strategies as well as oral health workforce and infrastructure.

## Background

Dental caries, the most common non-communicable disease worldwide, affects 60–90% of school children and the majority of adults. Oral health affects quality of life and is linked to overall health [1]. Oral health status information is essential to inform oral health education, prevention and disease management strategies [2]. The president of World Dental Federation (FDI) states:

Developing countries face great challenges in their quest for optimal oral care. Oral health is integral to general health and a basic human right and we must ensure cost-effective solutions become available to all. Promoting better research and obtaining valid data will help us achieve this objective. [3]

Rwanda, an East African nation of 11.3 million people, has made great progress in building its health care system [4]. The 1994 Genocide Against the Tutsi destroyed the human resource and physical infrastructure of Rwanda’s health and education sectors. In 2011 the Ministry of Health began a strategic initiative to build capacity and infrastructure for health care education and service delivery via the multi-sector Human Resources for Health (HRH) Rwanda program which supported the launch of the country’s first School of Dentistry (SoD) at the University of Rwanda (UR) in 2013 [5]. HRH Rwanda supports the World Health Organization (WHO) efforts on workforce development by responding to the global shortage of health workers, a challenge acutely problematic in sub-Saharan Africa, by building infrastructure and human resource capacity for health care education and service delivery. These efforts aim to address this critical shortage of trained health care providers [6].

Dental surgeons and dental therapists in Rwanda provide oral health care at district, provincial and referral government hospitals and at select private clinics. At the village level, government health posts provide analgesics for pain relief and referral to government hospitals or private clinics that offer dental care including the relief of pain and management of infection [7]. In 2011 the number of oral health professionals including dental assistants, therapists and surgeons was reported to be 122, for a provider to population ratio of approximately 1:92,000 [8]. Rwanda currently trains both dental surgeons and dental therapists at the University of Rwanda School of Dentistry, the only dental training institution in the country. The school is currently in the fifth year of implementing dental surgery training with an average intake of 22 dental surgery students per year. The dental therapy program, in existence since 1998, graduates an average of 25 dental therapists per year.

Historically, no national systematic assessment of oral health status in Rwanda had ever been conducted. The development phase of this first oral health assessment began in 2013, driven by three compelling reasons. First, since 2008 tooth and gum diseases have been the number one cause of morbidity in district hospitals [4]. Second, neighboring countries show a high prevalence of dental caries in young people aged 6–19 years: Uganda (80%, 2002), Tanzania (65%, 2004), Burundi (50.6%, 1988) and Democratic Republic of Congo (31%, 1982) [9]. Third, baseline oral health status information was needed to guide oral health capacity building. The decimation of oral health infrastructure in 1994, the undeveloped status of oral health research capacity, the scarcity of trained local human and adequate material resources, and the challenging geographical conditions necessitated an innovative approach to a systematic country-wide oral health assessment of Rwanda.

In 2013 Rwandan and international stakeholders representing oral health interest and expertise convened in Rwanda to provide input to the creation of the National Oral Health Survey of Rwanda (NOHSR). Integral to the stakeholder membership were representatives from the Ministry of Health Rwanda Biomedical Center Division of Non Communicable Diseases, the Rwanda Dental Surgeons Association and the Rwanda Dental (Therapists) Association, the University of Rwanda School of Dentistry, Tufts University School of Dental Medicine and Harvard School of Dental Medicine.

The aim of the first National Oral Health Survey of Rwanda was to establish efficient and practical methodologies for collecting oral health data and establishing a comprehensive database to: (a) assess national oral disease burden; (b) initiate national oral health surveillance; (c) inform strategies for oral health promotion, prevention and disease management; and (d) guide strategies for oral health workforce, infrastructure and educational development. This effort required the enhancement of oral health research infrastructure and the development of methods for systematic data collection, as well as the creation of operations and procedures for study implementation that were contextually appropriate for Rwanda. This paper describes the international collaboration that lead to the implementation of the first NOHSR and the findings for selected oral health indicators that provide baseline information regarding oral disease burden in Rwanda.

## Methods

A cross-sectional study was designed to obtain oral health data that would be representative of the country of Rwanda. A multi-method approach was used and included: (a) an interviewer-administered questionnaire to obtain demographic and self-reported oral health information, and (b) an oral health epidemiologic screening to observe and record key oral health indicators.

### Questionnaire

Questions and oral health indicators were selected from resources available from the World Health Organization and had been previously pilot tested and recommended for adaptation to local or national settings [10]. Additional guidance in the survey development was obtained from the Association of State and Territorial Dental Directors (ASTDD) Basic Screening Surveys [11,12].

The final survey instrument included a questionnaire evaluating: (a) demographics (17 closed-ended questions), (b) oral health practices and behaviors (19 closed-ended questions), and (c) oral health quality of life (8 closed-ended and Likert scale). Standard measures were used for assessing oral diseases and conditions [10–12] (Table 1). Age-appropriate and contextually relevant versions of the questionnaire and oral health screening forms were developed for children, adolescents and adults.

**Table 1.** Survey categories and selected variables.

Socio Demographic Characteristics (Questionnaire)
Sex
Age
Site
Location
Medical insurance
Dental insurance
Level of education
Occupation
Oral Health Indicators (Epidemiologic Screening)
Intraoral and extraoral lesions
Dentition status
Decayed, missing, filled primary and permanent teeth
Sealant status
Periodontal health
Prosthesis status
Associated dental conditions
Fluorosis
Dental treatment urgency
Oral Health Practices and Behaviors (Questionnaire)
Oral health practices
Oral health associated behaviors
Dental visits
Oral Health Quality of Life (Questionnaire)
Perceived state of health and oral health
Oro facial pain
Social effects

Questionnaires were translated into Kinyarwanda and back-translated at the University of Rwanda School of Public Health (UR SPH). The data collection tools were pretested in English at Tufts University School of Dental Medicine (Boston, USA) and in Kinyarwanda at the University of Rwanda School of Dentistry (Kigali, Rwanda) for face and content validity. All necessary adjustments were made to the data collection tools following the pretesting of the instruments.

Adult dental screenings recorded the decayed, missing and filled status of 28 teeth. Edentulism was defined as the complete absence of teeth (excluding third molars). Third molars, often difficult to visualize under field conditions of this study, were not included in these assessments. The cause of missing teeth was assumed to be caries since treatment histories were not available. Dental caries was recorded as a visually detectable cavitated lesion. Substantial oral debris was considered to be present when soft or hard deposits covered one-third or more of a tooth surface.

A Computer Assisted Personal Interview (CAPI) software system was designed at Tufts University specifically for electronic data collection for the NOHSR. The CAPI system was customized for skip patterns to accommodate child, adolescent and adult questionnaires. The dual language capabilities of the CAPI system provided the international team with simultaneous access to the questionnaires in English as well as Kinyarwanda with one keystroke.

### Sampling

Determination of sample size and selection of sample sites were based on the Pathfinder stratified cluster methodologies provided in WHO Oral Health Surveys – Basic Methods appropriate for national-level systematic studies and providing data suitable for planning and monitoring of oral health programs [10]. In consultation with the National Institute of Statistics Rwanda (NISR), 15 sample sites representing 4 provinces of Rwanda and the capital city of Kigali were randomly selected to reflect the country’s approximate urban/rural ratio (20%/80%) and provincial population distributions [13]. This resulted in 4 urban sites (2 urban sites in Kigali and 2 sites with large urban populations outside the capital city) and 11 rural sites.

The study population included five age groups (2–5, 6–11, 12–19, 20–39, and 40 and above years) consistent with population demographics for Rwanda and accepted oral health sampling domains [14]. According to the Pathfinder methodology, a sample of 25 subjects in each age group for each site was sufficient in populations where dental caries and periodontal disease levels are estimated to be low (greater than 50% caries free) [10]. A study conducted in Kigali found the prevalence of dental decay at 38.1% for 607 school-aged children (ages 6–16 years) [15]. A study from the Department of Dentistry at the former Kigali Health Institute reported 43% of study participants (n = 384) presented with gingivitis, 22.2% with early periodontitis, 8.3% with advanced periodontitis and 3.1% with juvenile periodontitis [16]. Based on this pilot data the sample size should be 25 individuals in each of 5 age groups at each site for a minimum sample size of 1875 participants [10]. Field logistics required periodic assessment of the number of individuals screened in each age group at each site. During data collection, if the number of individuals screened in an age group was noted to be greater than 25, the additional enrolled participants were included in our analysis. In 2016, the estimated life expectancy in Rwanda was 64 years. The population of Rwanda aged 65 years and above was estimated to be less than 3% thereby possibly precluding our ability to recruit the requisite number of participants in this age group [4]. Consequently, a separate age group for 65 years of age and older was not included.

### Calibration of examiners

All data collectors attended a three-day workshop with training and calibration sessions at the UR SoD. Data collection methods were standardized and training sessions were based on specific criteria outlined in a Manual of Procedures. The data collectors were calibrated against a gold standard examiner during calibration exercises. Additionally, each data collector was further calibrated against a gold standard examiner for an additional 20 screenings during the initial stages of the study period. Two gold standard examiners were available at all times to supervise data collection and assure accuracy of data entry. Recalibration exercises were held at the end of weeks one, two, and four during the six-week data collection period.

### Survey implementation

The survey was conducted during a six-week period in November and December 2016. The study was promoted and organized through local community leaders at the district, sector and cell levels. Study information was shared in community gathering locations and health clinics prior to, and on the day of, data collection. All promotion was by word of mouth means where community leaders informed the population. Individuals were randomly selected and notified of their selection to participate by the community health worker responsible for the selected site. Each participant was required to be a resident of the selected community.

The survey was conducted in Kinyarwanda at central locations in the community (local community facilities or health centers). Oral screenings consisted of visual examinations performed in artificial light (headlamps). A single field survey team collected the data throughout the entire data collection period. The data collection field team was comprised of seven dental therapist data collectors and two dentist research investigators. Data collectors used a structured interview process with objective indices and criteria to collect information and record oral diseases and conditions. Data were reviewed on a daily basis to ensure that all required information was recorded accurately and completely. The dentist research investigators were available on site at all times during data collection to monitor the activity and insure the quality of the data, coordinate multiple-computer data management, and provide support for equipment use, computer maintenance, and data backup.

### Methods of analysis

Data were entered directly into the CAPI system and analyzed using Statistical Package for Social Sciences (SPSS) Version 22, Stata version 13.1 (StataCorp LP, College Station, TX) and SAS version 9.4 (SAS Institute Inc., Cary, NC) for the selected variables. Descriptive statistics, including counts and percentages for categorical variables and means and standard deviations (SDs) for continuous variables, were calculated. Chi-square test was used to determine associations between categorical clinical outcomes and demographic variables of interest (age group, sex, education level, and location). Fisher’s exact test was used in place of the chi-square test to analyze categorical data with insufficient expected cell counts. Because continuous data were not normally distributed, the Mann-Whitney U-test and Kruskal-Wallis test were used to determine associations of continuous clinical outcomes and independent variables. Dunn’s test with Bonferroni correction was used for pairwise comparisons. Analyses of education level included data only from participants 20 years of age or older, due to the fact that personal information on education was only collected for this group.

### Ethical and government approvals

Ethical approval was obtained from the University of Rwanda College Medicine and Health Sciences on behalf of the Rwanda National Ethics Committee – 51/CMHS IRB/2016. The National Institute of Statistics of Rwanda (NISR) authorized the study and issued a study visa. Scientific Review approval was obtained from the National Health Research Committee of the Ministry of Health in Rwanda. Further authorizations to collect data were obtained from district mayors or sector leaders of the respective communities.

## Selected results

The NOHSR established the first comprehensive national oral database for planning and monitoring of activities by the Ministry of Health and the University of Rwanda School of Dentistry. Selected results are presented here to provide an overview of the oral health findings of the study and provide a snapshot of oral disease burden in Rwanda.

### Demographic variables

The study population consisted of 2097 participants (61.1% female) representing all provinces in Rwanda and the capital city Kigali. The mean (SD) age was 22.5 (19.6) years (range 2–104). In the age group 40 years and older, 170 individuals were between ages 40–49, 96 were between ages 50–64 and 95 were 65 years of age and older. Nearly three quarters (73.8%) resided in rural areas. For those ages 20 years and above, most (86.8%) completed primary school or less. The most often reported occupation of those 18 years of age and older was agriculture (68.5%). The majority of the study population (78.7%) reported having community based medical insurance coverage and over two-thirds (70.0%) reported having health coverage that covered dental treatment (Table 2).

**Table 2.** Socio-demographic information.

		Male n (%)	Female n (%)	Total n (%)	p-value
Variable					
Total participants		816 (38.9)	1281 (61.1)	2097 (100.0)	
Age (years)	2–5	206 (25.2)	194 (15.1)	400 (19.1)	<.0001
	6–11	201 (24.6)	224 (17.5)	425 (20.3)	
	12–19	164 (20.1)	247 (19.3)	411 (19.6)	
	20–39	127 (15.6)	318 (24.8)	445 (21.2)	
	40+	118 (14.5)	298 (23.3)	416 (19.8)	
Education*	None	55 (22.5)	209 (34.0)	264 (30.7)	<.0001
	Any primary (1°)	143 (58.4)	339 (55.1)	482 (56.1)	
	Some secondary (2°)	24 (9.8)	40 (6.5)	64 (7.4)	
	Secondary (2°) or more	23 (9.4)	27 (4.4)	50 (5.8)	
Location**	Urban	238 (29.2)	312 (24.4)	550 (26.3)	0.02
	Rural	578 (70.8)	969 (75.6)	1547 (73.8)	
Medical Coverage***	CBHI^§§^	648 (79.4)	1001 (78.3)	1649 (78.7)	0.41
	Other insurance	30 (3.7)	38 (3.0)	68 (3.3)	
	None reported	138 (16.9)	240 (18.8)	378 (18.0)	
Dental Insurance****	Yes	577 (72.7)	846 (68.1)	1423 (69.9)	0.03
	No	217 (27.3)	397 (31.9)	614 (30.1)	
Province	Kigali (2/0)^§^	117 (14.3)	159 (12.4)	276 (13.2)	0.71
	Southern Province (0/3)	161 (19.7)	250 (19.5)	411 (19.6)	
	Western Province (1/3)	214 (26.2)	347 (27.1)	561 (26.8)	
	Northern Province (1/2)	155 (19.0)	263 (20.5)	418 (19.9)	
	Eastern Province (3)	169 (20.7)	262 (20.5)	431 (20.6)	
Total		816 (100.0)	1281 (100.0)	2097 (100.0)	

*For those ≥20 years of age

**Urban/rural sites selected randomly by National Institute of Statistics Rwanda

***Total number of respondents was 2095

****Missing data represents 60 people who answered ‘I do not know’

^§^Number of urban sites/number of rural sites

^§§^Community Based Health Insurance

### Selected oral health indicators

The mean (SD) number of teeth for the adult population was 26.20 (3.27) (Table 5). No one in the study population was found to be edentulous. Nearly two-thirds (64.9%) of the study population had caries experience and 54.3% had untreated caries. Nearly three quarters (74.4%) of study participants over 20 years of age had caries experience and 60.7% had untreated caries. Caries experience varied significantly with age, level of education geographical location (p < .05). Untreated caries varied significantly with age, and geographical location (p < .05) but not by education level (p > .05) (Table 3).

**Table 3.** Caries experience and untreated caries by sex, age and location.

	Caries Experience (Overall DMFT*>0**)					Untreated Caries (D or d > 0)				
	Yes		No			Yes		No		p-value
Characteristic	n	%	n	%	p-value	n	%	n	%	
Total (n = 2097)	1362	64.9	735	35.1		1138	54.3	959	45.7	
Sex										
Male	531	65.1	285	34.9	0.93	439	53.8	377	46.2	0.73
Female	831	64.9	450	35.1		699	54.6	582	45.4	
Education***										
None	212	80.3	52	19.7	0.02	173	65.5	91	34.5	0.17
Any 1°	353	73.2	129	26.8		285	59.1	197	40.9	
Some 2°	41	64.1	23	35.9		34	53.1	30	46.9	
2° or more	34	68.0	16	32.0		28	56.0	22	44.0	
Age (years)										
2–5	251	62.8	149	37.3	<0.001	199	49.8	201	50.3	<0.001
6–11	241	56.7	184	43.3		239	56.2	186	43.8	
12–19	229	55.7	182	44.3		177	43.1	234	56.9	
20–39	300	67.4	145	32.6		250	56.2	195	43.8	
40+	341	82.0	75	18.0		273	65.6	143	34.4	
Location										
Urban	335	60.9	215	39.1	0.02	271	49.3	279	50.7	0.006
Rural	1027	66.4	520	33.6		867	56.0	680	44.0	

* T = permanent tooth/t = primary tooth/D = decayed permanent tooth/d = decayed primary tooth/M = missing permanent tooth/m = missing primary tooth

** dmft for ages 2–5/dftDFT for ages 6–11/DMFT for ages 12 and over

*** For those ≥ 20 years of age

**Table 4.** Mean number primary decayed (dt), filled (ft), and decayed/filled (dft) teeth in children aged 2–11* by age, sex and location.

Characteristic	dt		df		dft	
	Mean (SD)	p-value	Mean (SD)	p-value	Mean (SD)	p- value
Total (n = 407)	1.68 (2.53)		0.01 (0.09)		1.69 (2.53)	
Age, years						
2–5	1.82 (2.83)	0.89	0.01 (0.11)	0.22	1.83 (2.64)	0.89
6–11	1.55 (2.20)		0.00 (0.07)		1.55 (2.20)	
Sex						
Male	1.74 (2.65)	0.68	0.01 (0.10)	0.68	1.75 (2.65)	0.65
Female	1.62 (2.41)		0.01 (0.08)		1.63 (2.41)	
Location						
Urban	1.74 (2.39)	0.26	0.01 (0.12)	0.31	1.76 (2.42)	0.27
Rural	1.66 (2.58)		0.01 (0.08)		1.66 (2.57)	

*Primary teeth for those with at least 1 primary tooth

**Table 5.** Mean number of Teeth (T), Decayed Teeth (DT), Missing Teeth (MT) and DMFT adults 12 years of age and older* by age, sex and location.

	T		DT		MT		FT		DMFT	
Characteristic	Mean (SD)	p-value	Mean (SD)	p-value	Mean (SD)	p-value	Mean (SD)	p-value	Mean (SD)	p-value
Total (n = 1272)	26.20 (3.27)		1.36 (2.22)		1.80 (3.27)		0.02 (0.20)		3.19 (4.32)	
Age, years										
12–19	26.60 (3.18)	<0.001	0.67 (1.09)	<0.001	1.40 (3.18)	0.006	0.02 (0.27)	0.46	2.09 (3.24)	<0.001
20–39	27.02 (1.60)		1.37 (2.12)		0.98 (1.60)		0.03 (0.19)		2.38 (2.98)	
40+	24.92 (4.17)		2.04 (2.85)		3.08 (4.17)		0.02 (0.15)		5.13 (5.61)	
Education***										
None	25.10 (4.01)	<0.001	2.11 (2.97)	0.002	2.90 (4.01)	0.001	0.01 (0.09)	0.29	5.02 (5.41)	0.001
Any 1°	26.25 (3.05)		1.61 (2.42)		1.75 (3.05)		0.02 (0.18)		3.39 (4.43)	
Some 2°	27.09 (1.39)		0.98 (1.37)		0.90 (1.39)		0.03 (0.18)		1.92 (2.27)	
2° or more	27.18 (1.27)		1.18 (1.52)		0.82 (1.27)		0.06 (0.31)		2.06 (2.49)	
Sex										
Male	26.27 (3.27)	0.25	1.26 (2.55)	0.07	1.73 (3.27)	0.25	0.02 (0.16)	0.91	3.01 (4.38)	0.02
Female	26.16 (3.27)		1.41 (2.04)		1.84 (3.27)		0.02 (0.22)		3.27 (4.29)	
Location										
Urban	26.80 (2.64)	<0.001	0.97 (1.67)	<0.001	1.20 (2.64)	<0.001	0.05 (0.35)	0.03	2.22 (3.42)	<0.001
Rural	25.98 (3.55)		1.50 (2.37)		2.02 (3.44)		0.01 (0.11)		3.53 (4.55)	

* Permanent teeth for those with at least one permanent tooth

The overall mean (SD) number of decayed (d) filled (f) and primary teeth (t) for ages 2–11 years was 1.69 (2.53). The mean dt (1.68, SD 2.53) contributed more to dft than the mean ft (0.01, SD 0.09) (Table 4). Of those 12 years of age and older, the mean decayed (D), missing (M) and filled (F) status of permanent teeth (T) was reported. The mean (SD) DMFT was 3.19 (4.32). The mean number of decayed teeth (DT) (1.36, SD 2.22) and the mean number of missing teeth MT (1.80, SD 3.27) contributed the most to DMFT and the mean number of filled teeth (FT) contributed the least (0.02, SD 0.20). For those 40 years of age and above, the mean (SD) for DT, MT, and DMFT was 2.04 (2.85), 3.08 (4.17), and 5.13 (5.61) respectively. The indicators for dental caries in this group were significantly greater (p < .05) than those aged 20–39 years except for FT. The number of T, DT, MT and DMFT varied significantly by age, education level and geographical location (p < .05) (Table 5).

Among adults 20 years of age and older, 32.4% had substantial oral debris and 60.0% had calculus. Calculus was reported in 69.7% of those aged 40 years and over. Substantial oral debris and calculus varied significantly by age, education level and geographical location (p < .05).

Less than 5% of the study population was reported to have evidence of dental trauma (2.0%), dental abscess formation (2.0%) and suspicious intra oral lesions (1.3%) and extra oral lesions (3.0%). Early dental care (within several weeks or months/including scaling as needed) was indicated for 61.3% of the population while immediate treatment (urgent relief of pain or infection) was required by 5.4%.

### Oral health quality of life and practices

Of the participants 63.3% self-reported their oral health to be fair and 15.3% reported their oral health to be poor. Nearly one third (32.5%) of those 40 years and above self-reported their oral health to be poor.

Nearly two-thirds of the study participants (63.9%) reported painful aching in the mouth during the 12 months preceding the study. This percentage was over three-quarters (76.8%) among those aged 20–39 years and nearly 90% among those aged 40 years and above. Respondents reported oral health quality of life challenges due to their mouth, teeth or dentures. Over half (54.6%) of those aged 20–39 years and nearly two-thirds (66.4%) of those 40 years of age and above reported difficulty doing usual jobs because of their mouth, teeth or dentures. Difficulty chewing was reported by over three-quarters (75.3%) of those 40 years of age and older. Over one-third (36.2%) of the participants reported being self-conscious or embarrassed because of their teeth or mouth. Data reported on quality-of-life indicators varied significantly by sex, age, education level and geographical location (p < .05), with the exception of pain which did not vary significantly with location or education level, and difficulty speaking which did not vary significantly with education level (p > .05). (Table 6).

**Table 6.** Self-report on quality of life by sex, age and location.

		Difficulty doing usual jobs* (n = 2093) n (%)	p-value	Difficulty chewing (n = 2088) n (%)	p-value	Self-conscious** (n = 2085) n (%)	p-value	Difficulty speaking (n = 2086) n (%)	p-value	Pain*** (n = 2097) n (%)	p-value
Sex	Male	231 (28.4)	<.0001	290 (35.8)	<.0001	242 (29.9)	<.0001	127 (15.7)	0.0003	480 (58.8)	0.0001
	Female	509 (39.8)		592 (46.4)		513 (40.2)		283 (22.2)		860 (67.1)	
Education											
	None	175 (66.5)	0.02	189 (72.1)	0.04	173 (65.8)	0.007	108 (40.9)	0.19	220 (83.3)	0.83
	Any 1°	285 (59.3)		319 (66.3)		281 (58.7)		171 (35.6)		395 (82.0)	
	Some 2°	30 (46.9)		36 (56.3)		28 (43.8)		18 (28.1)		53 (82.8)	
	2° or More	27 (54.0)		29 (58.0)		26 (52.0)		16 (32.0)		39 (78.0)	
Age (years)	2–5	43 (10.8)	<.0001	72 (18.1)	<.0001	55 (13.9)	<.0001	22 (5.6)	<.0001	145 (36.3)	<.0001
	6–11	86 (20.2)		111 (26.2)		87 (20.6)		36 (8.5)		233 (54.8)	
	12–19	93 (22.7)		125 (30.5)		104 (25.4)		38 (9.3)		254 (61.8)	
	20–39	243 (54.6)		263 (59.1)		227 (51.1)		139 (31.2)		342 (76.9)	
	40+	275 (66.4)		311 (75.3)		282 (68.3)		175 (42.2)		366 (88.0)	
Location	Urban	144 (26.2)	<.0001	179 (32.7)	<.0001	159 (29.0)	<.0001	78 (14.3)	0.0002	346 (62.9)	0.57
	Rural	596 (38.6)		703 (45.6)		596 (38.8)		332 (21.6)		994 (64.3)	
Total		740 (35.4)		882 (42.2)		755 (36.2)		410 (19.7)		1340 (63.9)	

* Difficulty doing usual jobs, attending school or participating in social activities

**Self-conscious or embarrassed

***Pain in the mouth during the 12 months preceding this survey

Even with the high prevalence of dental pain and access to medical and dental insurance, the majority of those participating in the study (70.6%) never visited an oral health provider for treatment. Of those who visited a dental practitioner, nearly all (98.7%) sought care because of pain. Of those who responded to the question why they were unable to access care, over half (60.3%) reported that cost was the major reason for not receiving care.

## Discussion

This study was a cooperative effort by the University of Rwanda Schools of Dentistry and Public Health and Tufts University and Harvard University Schools of Dental Medicine. From 2013 to 2016 a practical, efficient and cost-effective study design and innovative data collection methodology was developed. The close collaboration between Rwandan and US health and research professionals during the study design and implementation served to improve in-country oral health research capacity.

Strengths of this study include the large sample size representing the major geographic areas and age groups in Rwanda. The Pathfinder methodology for oral health surveys, advocated by the WHO for national-level systematic studies, provided the data collection approach suitable for planning and monitoring of oral health programs. The CAPI system allowed standardized and efficient collection of data via questionnaire and screening methodologies customized for contextual relevance in Rwanda. Sampling representation of the capital city (Kigali), urban sites outside the capital city, and rural sites in the four provinces closely mirrors the national population distribution [11]. Our sample participants reported that 86.8% of those at least 20 years of age had completed primary school or less which is consistent with national data [17]. Approximately three quarters of Rwandans engage in agriculture as their main occupation [18]. The majority of study participants (68.5%) reported that agriculture was their main occupation. Study participants reported 78.7% were covered by community based health insurance (CBHI). This is consistent with the national reported coverage of 74% [19].

The customized CAPI (computerized) data collection system developed at Tufts University School of Medicine was created to minimize data entry time, minimize data entry errors, and provide consistent questionnaire delivery and oral screenings. Features of the CAPI system include the ability to provide direct data entry, simultaneous access to dual language forms of the survey instrument, easy uploads of the data in text delineated files to analysis programs, skip patterns to accommodate child, adolescent and adult versions of the survey instruments, and easy daily assessment of data integrity. The CAPI survey instrument withstood the logistical challenges of field conditions and was readily adopted by the Rwandan data collectors. This design drove down the overall cost of the survey while enabling the collection of robust and meaningful data. The CAPI system provided an approach to oral health data collection appropriate and accessible for future oral health assessments in Rwanda. The unique feature of the CAPI oral health survey system of adaptability to a specific language provides for its potential use in other countries and localities.

Assessing the prevalence of periodontal disease is challenging for all populations. A variety of case definitions for periodontal diseases have been cited in the literature for population-based studies [20]. Designing a periodontal disease assessment method for this type of field study was difficult. We chose to use the presence of calculus and the presence of oral debris as clinical observations consistent with gingival and periodontal diseases [21,22].

The participants’ quality of life in performing usual daily activities and chewing was negatively impacted by adverse dental conditions. More than half of the participants aged 20 years and above felt self-conscious because of the teeth or mouth. The oral health care system was accessed almost exclusively for pain relief, not for preventive, restorative or non-emergent conditions. Consistent with this finding was the low number of filled teeth among the study population and low access to dental providers. Cost was reported as a major barrier to dental care despite nearly 70% of the population reportedly having insurance coverage that includes dental services. The challenging geographical terrain and climate, the relatively recent development of oral health workforce, the lack of oral health infrastructure and promotion programs, and the distribution of a limited number of oral health providers are considered factors that may influence the awareness and utilization of oral health services by the population. Given that associations between oral diseases and certain systemic diseases and disorders have become more evident, establishing this baseline information to identify the extent of oral disease prevalence was essential to begin to address oral health concerns, develop oral health strategies and integrate efforts on non-communicable disease prevention and promotion in Rwanda [23,24].

The methodology for this study was not without limitations. Self-reported information in the survey was subject to responders’ interpretation of the question and social acceptability bias. The survey was conducted in predetermined locations in the community that possibly excluded those who were unable to reach the identified sites. In some locations, local study coordinators reported that men were less available than women due to work and travel obligations on the day of the survey. Nonetheless, the large sample size, the representative national sampling and the randomization efforts of site selection by the NISR and the community health workers in their recruitment of participants, make the database an invaluable resource for a national assessment of oral health in Rwanda.

Data-driven solutions to global burden of oral disease require the development of research infrastructure to inform strategic planning and the reimagining of the global dental workforce wherever access to dental education and dental providers is limited [25]. In-country leadership for the study included local faculty at the UR SoD and UR SPH who now have experiential knowledge to guide future periodic surveillance of the country’s oral health status and to educate future oral health researchers. Seven Rwandan dental therapists were trained and calibrated and now have experience in oral health data collection. They exist as a resource for continued national oral health surveillance and the education and training of oral health research workforce. The creation of the CAPI data collection instrument and the training of Rwandan oral health research personnel elevated the oral health research capacity to support evidence based strategies for oral health workforce and infrastructure development. Ultimately this serves to reduce the burden of oral diseases in Rwanda.

The ability to compare our selected results directly with findings from other studies in African countries is limited by variations in study designs. A study of periodontal disease in Zimbabwe reported the prevalence of calculus among 35–44-year-olds is 60% [26]. In South Africa, the caries prevalence of 4–6-year-olds living in Johannesburg was reported as 49.2% [27]. DMFT scores from Malawi, Uganda and Namibia for 12-year-olds ranged from 0.40 to 1.74, and for 15-year-olds in Malawi and Namibia they ranged from 0.71 to 3.05 [28–30]. Our study population demonstrated a similar or higher disease burden in each category. Although our study included 151 participants between aged 50–64 years and 95 of those were 65 years of age and older, none were edentulous. The absence of edentulism in the study population may be a reflection of access (or non-access) to dental services and may not reflect the burden of oral disease.

## Conclusion

The data generated from this study supports that Rwanda is challenged by a high burden of oral diseases. Nearly two-thirds of the study participants had caries experience and over 50% had untreated caries. Among adults 20 years of age and older, 32.4% had substantial oral debris and 60.0% had calculus. Quality-of-life challenges due to oral diseases/conditions including pain, difficulty chewing, self-consciousness, and difficulty participating in usual activities was reported by 63.9%, 42.2% 36.2%, 35.4% of participants respectively. In addition to improving oral health research capacity and providing national oral health baseline data, the first National Oral Health Survey of Rwanda created a database of oral health information that can be analyzed to provide critically valuable information for the development of effective oral health education, prevention and disease management strategies as well as oral health workforce and infrastructure development. This is essential to formulate and implement data-driven policies as well as support future research efforts for oral health improvement.

## Paper context

Rwanda, disproportionately affected by oral diseases and oral health workforce shortages, lacked oral health data and surveillance to inform strategies to address these challenges. Prior to this survey, no national oral health data were available. Through international collaboration, infrastructure and a replicable cost-effective study design were developed to build research capacity. The first national oral health survey was implemented and findings can inform future evidenced based policies, workforce development and methodologies for suitable oral health surveillance.
